# Predicting carbohydrate quality in a global database of packaged foods

**DOI:** 10.3389/fnut.2025.1530846

**Published:** 2025-03-12

**Authors:** Eric Antoine Scuccimarra, Alexandre Arnaud, Marie Tassy, Kim-Anne Lê, Fabio Mainardi

**Affiliations:** ^1^Nestlé Institute of Health Sciences, Nestlé Research, Société des Produits Nestlé, Lausanne, Switzerland; ^2^Division of Human Nutrition and Health, Wageningen University & Research, Wageningen, Netherlands

**Keywords:** food supply, free sugar, missing value imputation, carbohydrate quality, machine learning, added sugar, food labeling, nutritional databases

## Abstract

**Background:**

Carbohydrates are the major contributor to the energy intake of worldwide population. There is established evidence of links of carbohydrate quality with human health. Knowledge of specific carbohydrate in packaged food, such as added and free sugars, could help further investigate this link, however this information is generally not available.

**Objective:**

To develop an algorithm to predict the content of free sugars in a global database of packaged foods and beverages; and test the applicability of the algorithm to assess carbohydrate quality in packaged food products from different countries and monitor the evolution over time. Carbohydrate quality was defined using a 10:1|1:2 ratio for carbohydrate, fibers and free sugar, i.e., for every 10 g of total carbohydrates in a diet or product, there is at least 1 g of dietary fibers, and less than 2 g of free sugars for every 1 g of dietary fibers.

**Methods:**

We used a machine learning approach to predict added and free sugars, which enabled us to predict the carbohydrate quality of products from a global database of packaged food. Our predictions were tested by splitting the dataset into training, validation, and test sets, using US data.

**Results:**

We were able to predict free sugars and carbohydrate quality for 424,543 products in the U.S. and in 14 countries. The overall mean absolute error on the test set was 0.96 g/100 g of product. The predictions generalized with a high accuracy to non-US countries, and we were able to effectively predict the proportion of products meeting the 10:1|1:2 criteria in the food supply of 15 countries.

**Conclusion:**

Our methodology achieved high accuracy and is fully automated; it may be applied to other databases of packaged products and can be easily applied for continuous monitoring of the carbohydrate quality of the global supply of packaged food.

## Introduction

Carbohydrates are the primary energy source in the human diet, accounting for about 70% of the daily energy intake worldwide (1), with disparities between geographies (2). Quality is equally as important as the quantity of carbohydrates consumed, and several previous studies have provided evidence that links carbohydrate quality with human health (3–13). Carbohydrate quality can be measured through different proxies (14). One of those considers the ratios between carbohydrates, fibers and free sugars, commonly referred to as the “carbohydrates quality ratio” (15). In brief, it states for every 10 g of total carbohydrates in a diet or product, there is at least 1 g of dietary fibers, and less than 2 g of free sugars for every 1 g of dietary fibers (15). This ratio was defined as an interpretation of dietary recommendations, with a recommended consumption of 25–30 g of fibers, and free sugars limited to <10% of energy (1). The term free sugar in this paper follows the updated WHO definition and refers to all monosaccharaides and disaccharides added to foods and beverages by the manufacturer, or consumer, and sugars naturally present in honey, syrups, fruit juices, and fruit concentrates (16). The scientific rationale for this ratio is to favor consumption of fiber-rich foods, while limiting free sugars consumption, following WHO recommendations. This metric has proven to be helpful to identify products with higher nutritional value (15, 17) in particular with higher levels of protein, fiber, iron, magnesium, zinc, potassium, selenium, and vitamins B1, B3, and B9 as well as lower levels total sugars, free sugars, saturated fat, trans fat and cholesterol. Moreover, it has been modelled that individuals consuming higher proportions of products meeting this carbohydrates ratio would have higher diet quality, as well as lower risk factors for cardiometabolic diseases (7, 18). However, identifying the “free sugars” content of packaged food products can be challenging, which may currently limit the applicability of this metric. Indeed, while the content of “total sugars” is typically part of the mandatory food nutritional fact declaration, only a few countries, including the USA, Mexico and Brazil, have made it mandatory to declare “added sugars” (19). We follow the FDA’s definition of added sugars: caloric sweeteners that are added to foods as ingredients during food processing, during food preparation, or at the table (20). Sugars naturally present in milk and fruit are not added sugars, by definition.

Therefore, in most countries, the content of added and free sugars in packaged foods can only be inferred from the information on pack, namely the nutrition facts and the list of ingredients. Several methodologies have been developed previously to overcome the lack information on free- or added sugars. For non-packaged foods, free sugars may be estimated from *a priori* knowledge, for example the food group (e.g., raw vegetables), or from a typical recipe in the case of a composite packaged food product (21–23). This approach, however, relies on a number of manual and somewhat subjective steps. An alternative method based on automated imputation algorithm and machine learning algorithm has been applied to the Philippines’ food composition table (24); this method used the information available in such composition tables: names the food groups and the nutritional composition. However, for packaged foods the ingredient list is usually available and can be usefully incorporated into a predictive model.

Machine learning methods with the potential to extract patterns from large amounts of data and make reliable predictions that require no manual or *ad hoc* calculations have been proposed. Davies et al. (25) developed a machine learning approach that can predict the added-sugar content of packaged foods and beverages, trained on a large US dataset (US Label Insight) that contains information on the characteristics of products and their added-sugar content. A similar study (26) applied an XGBoost model to a U.S. subset of another commercial database (WW International Inc.). However, it was acknowledged that the applicability of their algorithm to other countries remained unknown. Given our interest in making predictions on a global dataset, we needed to create a predictive model from the ground up instead of relying on any of the existing published methods.

The purpose of this study is twofold: first, we developed an algorithm to predict the content of free sugars in a global database of packaged foods and beverages; second, we tested the applicability of our algorithm to track the carbohydrate quality ratio in packaged food products from different countries and monitor the evolution over time.

## Methods

### Selection of the database

The Mintel Global New Products Database (GNPD) is an online database created and maintained by Mintel, a private international market research company. The GNPD consists of around 130,000 new products each year compiled by trained shoppers monitoring product launches in 86 countries and provides an up-to-date representation of the global food supply (9). The database includes products from 24 distinct categories of packaged foods, with fresh and whole foods, such as fruits, not usually included. The database includes information available on-pack, including nutrient content and the ingredient list. We considered products launched from January 2014 to February 2024, representing a total of 2,412,463 products. Data cleaning steps are detailed in Supplementary material. A total of 887,575 products were kept (36.8% of raw dataset) including 123,035 products declaring added sugars (80.7% of initial products declaring added sugars before cleaning).

### Prediction of free sugars

**Figure 1:**
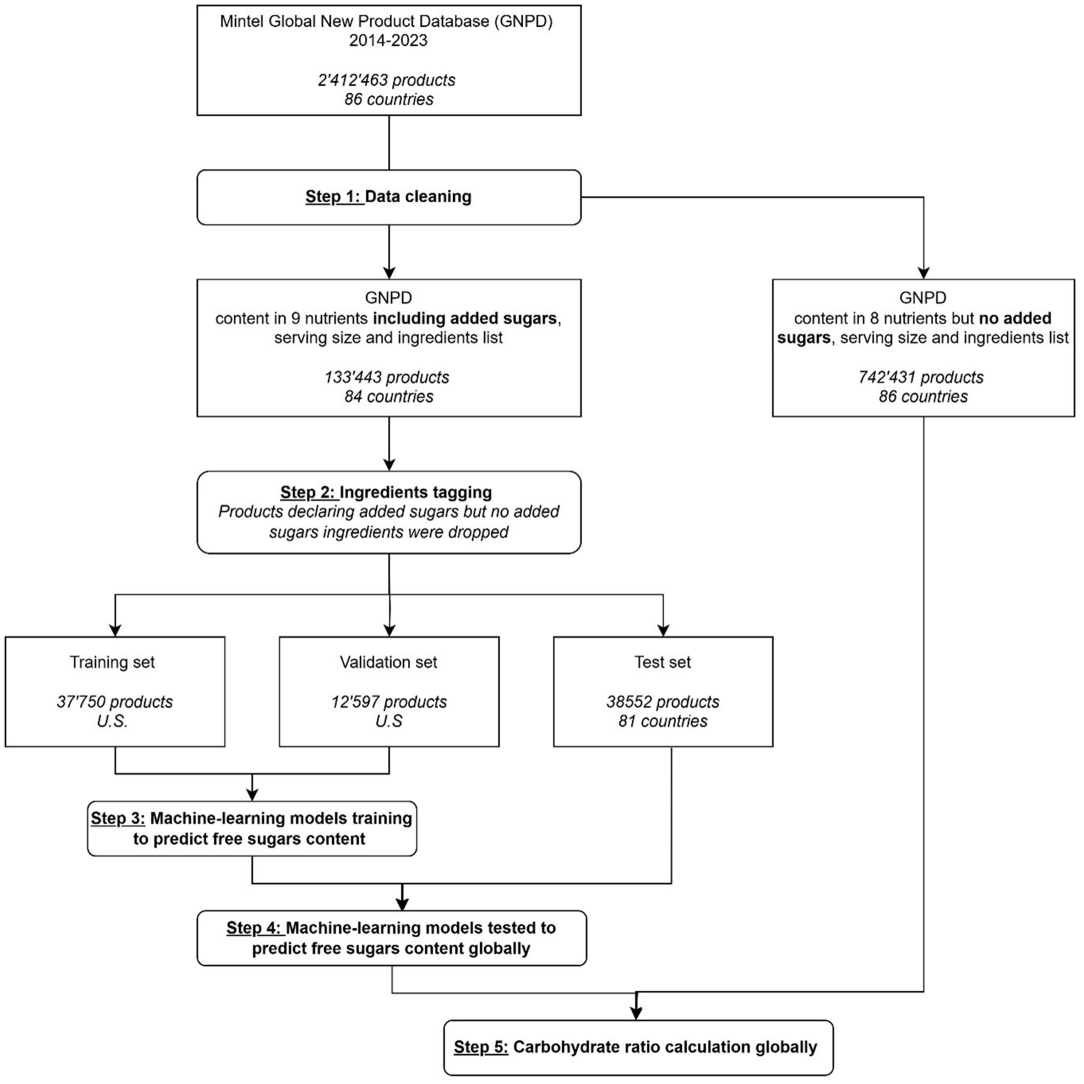
Flow diagram presenting the five steps of the automated methodology developed to predict free sugars and calculate carbohydrate ratio.

**Figure 2:**
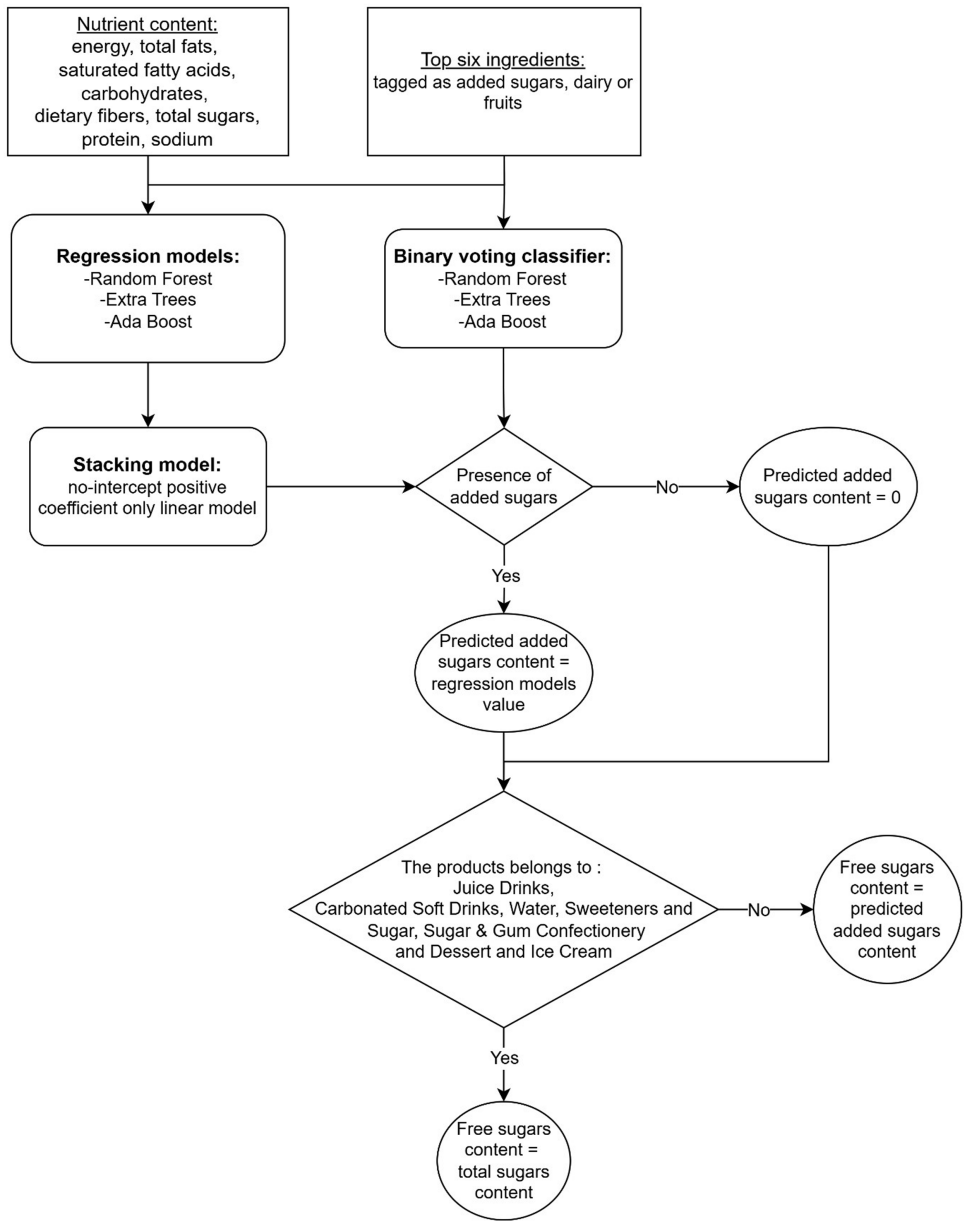
Flow diagram of the components of the machine learning model for the prediction of free sugars content in packaged food supply databases.

The automated methodology to predict free sugars was developed in steps 1 to 4 presented in Figure 1, which includes information about the datasets used at each stage. After data cleaning (step 1), the Mintel GNPD featured 887,575 products with complete information available on-pack (seven nutrients and a valid serving size/measure), launched between January 2014 and February 2024. For each product, ingredients were tagged as added or naturally occurring sugars (dairy or fruits/vegetables) (step 2). The machine learning models were trained on a U.S. GNPD subset (training & validation sets) (step 3) and then tested on products from 81 other countries (step 4). The development of machine learning models to predict free sugar content is outlined in Figure 2, while more detailed explanations can be found on each step of the methodology in the Supplementary material. Briefly, the machine learning models use as features the first six ingredients of each product, which were tagged as added sugars, dairy or fruits and the content of energy, total fats, saturated fatty acids, carbohydrates, dietary fibers, total sugars, protein, and sodium, all per 100 g. Two types of machine learning models were built: three binary classifiers to evaluate the presence of added sugars in a product and, if present, stacked tree-based regression models to predict the content in added sugars quantitatively. Predicted added sugars content was considered as a good approximation for free sugars for all products except if they belonged to the following categories: “Juice Drinks,” “Carbonated Soft Drinks,” “Sweeteners & Sugar,” and “Sugar & Gum Confectionery”; and the following sub-categories:” Flavoured Water,” “Honey,” “Syrups,” “Ready To Drink (Iced) Tea,” and “Water Based Ice Lollies, Pops & Sorbets.” For these categories/subcategories, we considered total sugars as a good approximation for free sugars. We chose to train our algorithm using products from the USA, which represents over 56% of the total products with declared added sugar from the cleaned dataset as it can be considered a reliable source of information due to the requirement for added sugars to be declared on pack.

### Definition and calculation of carbohydrate quality metric

The machine learning models were applied to the 742,431 products declaring serving size, ingredient list and content in seven nutrients on-pack but not declaring added sugars to predict carbohydrate ratio (total carbohydrates/free sugars/dietary fibers) globally (step 5 in Figure 1).

Using either the declared added sugar content as an estimation for free sugars or our predicted free sugar content, we identified the products in the database meeting the following criteria for carbohydrate quality (10:1|1:2): for every 10 g of total carbohydrates in a diet or product, there is at least 1 g of dietary fibers, and less than 2 g of free sugars for every 1 g of dietary fibers. The carbohydrates/fiber ratio was based on recommendations from the American Heart Association (27). The rationale for the fiber/free sugar was detailed in other articles (15).

Following previous carbohydrate quality assessment (12), performance of the algorithm in predicting the percentage of products meeting the 10:1|1:2 criteria was assessed from 2014 to 2024 for 18 categories: Bread & Bread Products, Cakes, Pastries & Sweet Goods, Coffee and Tea, Cold Cereals, Flavoured Milk, Fruit Snacks, Fruit/Flavoured Still Drinks, Hot Cereals, Instant Noodles, Instant Pasta & Rice, Juice, Malt & Other Hot Beverages, Pizzas, Plant Based Drinks (Dairy Alternatives), RTDs, Savoury Biscuits/Crackers, Snack/Cereal/Energy Bars, Sweet Biscuits/Cookies. Furthermore, the performance in the algorithm was assessed in 14 countries, other than the U.S., selected based on the following criteria: each world region must be represented, countries having implemented a sugar-reduction tax policy should be included, and countries with the largest sample sizes are prioritized. The list of selected countries was the following: Australia, Brazil, Chile, France, Germany, India, Malaysia, Mexico, Nigeria, Philippines, Singapore, South Africa, Thailand, UK. In each category or country, the percentage of products meeting the 10:1|1:2 ratio using true or predicted value were compared with proportion *z*-tests. Finally, the predictions of carbohydrate quality were used to monitor evolution in the carbohydrate quality in each category and country from 2014 to 2024.

All calculations and visualizations were done with Python 3.9 and R 4.2.3, with scikit-learn used for machine learning.

## Results

### Performance of the algorithm in predicting free sugars and carbs ratio in the U.S. from 2014 to 2024

**Figure 3:**
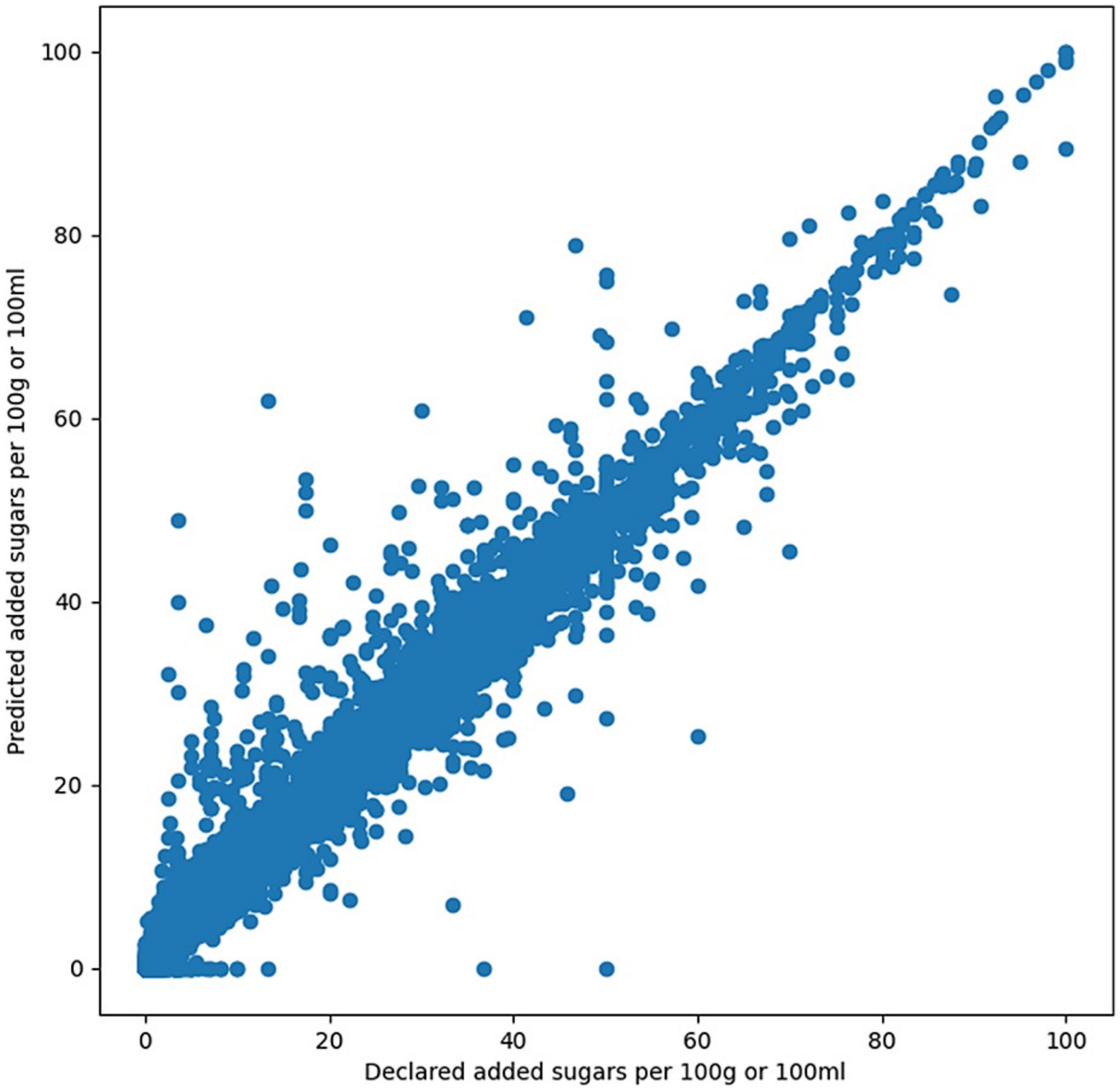
Each dot represents a product from the US validation set (*N* = 12,597).

The performance of the machine learning models in predicting free sugars was visualized using a scatter plot (Figure 3). The overall mean absolute error (MAE) on the test set was 0.96 g/100 g and the R^2^ between predicted and declared values was 0.98. Inspection of residuals did not show evidence of a systematic bias (under or over-estimation). Applying the kNN (“k-Nearest Neighbors”) algorithm as in a previous study (10) to our dataset, using the same nutrients as our models and omitting starch as it is typically not declared on labels, resulted in an MAE of 2.51. Further information comparing the performance of our method and the kNN method is in Supplementary Table 4. Our most accurate predictions, by mean absolute percentage error (MAPE) were the following categories: “Sugar & Gum Confectionery” (MAE = 2.18, *R*^2^ = 0.92, MAPE = 3.8%), and “Breakfast Cereals” (MAE = 0.77, *R*^2^ = 0.98, MAPE = 4.2%), while it was the least accurate for “Meals & Meal Centers” (MAE = 0.45, *R*^2^ = 0.86, MAPE = 24.9%), and “Soup” (MAE = 0.35, *R*^2^ = 0.66, MAPE = 36.3%). We find MAPE to be a more useful metric for ranking performance on certain categories as categories with little added sugar tend to have low MAE, and vice versa. Additionally, there were some notable errors where the model predicted no added sugars when the product actually contains more than 10 grams of added sugars. In all these cases, no added sugar ingredients were identified by our ingredient tagging methods. We found that the products with the largest absolute errors tended to have discrepancies between the declared added sugars and the ingredients, i.e., they either declared high amounts of added sugars with no added sugar ingredients or vice versa.

The predicted proportion of products meeting the carbohydrate ratio was a fair estimate of the “true” proportion, calculated from the declared added sugars values in the U.S. Differences between the predicted and true proportions were not statistically different in any of the 18 categories evaluated (Table 1). For Flavoured Milk, the proportion was 0% in both cases and a *p*-value could therefore not be calculated.

In the U.S. packaged food launched from 2014 to 2024, the percentage of packaged products meeting carbohydrates ratio ranged from 60% in Hot Cereals (i.e., cereal that must be cooked before eating, including oatmeal, instant oatmeal, hot wheat, and other grain products), to 0% in Flavoured Milk and Malt & Other Hot Beverages. The second and third categories in terms of number of products meeting carbohydrate ratios were grain- or fruit based: Snack/Cereal/Energy Bars (44%) and Fruit Snacks (42%). In contrast, three categories with less than 1 percent of products meeting the 10:1|1:2 criteria were liquids: Juice, Flavoured Milk, and Malt & Other Hot Beverages.

**Table 1:** Predictions on US data, years 2014–2023.

Category	*n*	Carbohydrates (g)	Fibers (g)	Free sugars (g)	% Products meeting 10:1 1:2 ratio (true)	% Products meeting 10:1 1:2 ratio (predicted)	*p*-value
Bread & Bread Products	401	48.6 ± 8.9	3.6 ± 3.1	4.1 ± 4.1	21.4	21.4	1.00
Cakes, Pastries & Sweet Goods	433	53.2 ± 11.9	1.7 ± 2.1	26.0 ± 12.2	3.7	3.5	0.85
Coffee and Tea	30	38.8 ± 33.5	3.1 ± 7.3	17.9 ± 20.3	16.7	16.7	1.00
Cold Cereals	485	71.1 ± 17.0	7.4 ± 3.7	20.1 ± 11.1	32.0	33.4	0.63
Flavoured Milk	19	9.5 ± 2.6	0.3 ± 0.5	4.3 ± 1.8	0.0	0	NA
Fruit Snacks	305	65.8 ± 24.7	6.4 ± 5.1	11.1 ± 18.8	42.0	41.3	0.87
Fruit/Flavoured Still Drinks	38	9.2 ± 4.8	0.1 ± 0.4	8.4 ± 5.0	5.3	5.3	1.00
Hot Cereals	124	66.0 ± 13.6	8.8 ± 2.6	10.2 ± 10.9	59.7	60.5	0.90
Instant Noodles	73	59.4 ± 15.0	2.7 ± 1.5	2.9 ± 2.9	4.1	4.1	1.00
Instant Pasta & Rice	21	61.8 ± 15.1	3.7 ± 3.3	0.6 ± 1.8	9.5	9.5	1.00
Juice	104	8.4 ± 3.8	0.3 ± 0.6	6.9 ± 3.5	1.0	1.0	1.00
Malt & Other Hot Beverages	67	73.0 ± 14.1	4.1 ± 3.5	54.3 ± 16.5	0.0	1.5	0.32
Pizzas	146	26.5 ± 4.8	1.5 ± 0.6	1.1 ± 1.2	5.5	5.5	1.00
Plant Based Drinks	94	5.3 ± 7.6	0.7 ± 1.8	2.0 ± 2.9	34.0	36.2	0.76
RTDs	108	5.7 ± 4.6	0.2 ± 0.4	3.7 ± 3.6	12.0	11.1	0.83
Savoury Biscuits/Crackers	229	66.1 ± 11.7	4.6 ± 4.9	4.0 ± 5.0	24.0	24.0	1.00
Snack/Cereal/Energy Bars	466	52.9 ± 14.3	8.6 ± 6.0	14.6 ± 11.8	44.6	45.5	0.79
Sweet Biscuits/Cookies	767	64.7 ± 9.5	3.0 ± 2.8	30.4 ± 10.7	5.3	4.6	0.48

Values for carbohydrates, fibers and free sugars are as declared. The last three columns compare, for each category, the proportion of products meeting the 10:1/1:2 ratio, when using the declared values for free sugars (true) and the predicted values (predicted). *p*-values refer to a *z*-test to compare proportions, *p* > 5% means that the difference between the proportions is not statistically significant. “NA” for Flavoured Milks means that the *p*-value could not be calculated.

### Generalization to other countries

While the machine learning models were solely trained on U.S. data, predictions in 14 other countries were generally very accurate (Table 2). The biggest discrepancy was found for France, where the declared values gave 20% of products meeting the ratio (aggregating all categories), while the predicted percentage was 26.7, even though the difference was not found to be statistically significant.

**Table 2:** Predictions of ratios for selected countries, aggregated across all the categories in Table 1.

Market	*n*	Carbohydrates (g/100 g)	Fibers (g/100 g)	Free sugars (g/100 g)	% Products meeting 10:1 1:2 ratio (true)	% Products meeting 10:1 1:2 ratio (predicted)	*p*-value
Australia	145	28.2 ± 23.2	3.4 ± 5.1	8.5 ± 8.9	31	30.3	0.90
Brazil	904	51.4 ± 20.0	3.4 ± 3.5	14.9 ± 14.3	20.5	20.5	1.00
Chile	11	52.1 ± 27.2	3.5 ± 3.5	16.9 ± 16.6	18.2	18.2	1.00
France	15	46.7 ± 26.1	5.1 ± 5.6	10.0 ± 9.6	20	26.7	0.67
Germany	20	42.0 ± 18.8	9.2 ± 6.6	10.8 ± 10.0	50	45	0.75
India	1,191	57.5 ± 22.9	5.4 ± 5.7	10.6 ± 12.9	32.2	32	0.90
Malaysia	256	57.6 ± 22.2	3.3 ± 3.1	13.2 ± 12.0	9.8	9.4	0.88
Mexico	3,497	49.7 ± 26.8	3.4 ± 4.5	16.8 ± 15.7	15.7	16	0.69
Nigeria	267	62.9 ± 18.3	4.6 ± 5.1	17.9 ± 14.3	22.1	22.8	0.84
Philippines	302	53.6 ± 28.1	3.7 ± 5.3	18.2 ± 16.6	10.9	12.3	0.61
Singapore	395	48.4 ± 28.1	3.5 ± 5.1	14.3 ± 15.1	13.9	12.9	0.68
South Africa	87	58.2 ± 20.1	4.5 ± 5.7	20.9 ± 13.8	18.4	18.4	1.00
Thailand	67	51.6 ± 29.3	5.1 ± 4.8	7.2 ± 8.9	25.4	26.9	0.84
UK	27	53.8 ± 24.2	12.7 ± 9.2	11.6 ± 16.8	66.7	66.7	1.00
USA	3,910	54.4 ± 22.6	4.4 ± 4.5	16.5 ± 15.6	21.2	21.3	0.89

Values for carbohydrates, fibers and free sugars are as declared. The last three columns compare, for each category, the proportion of products meeting the 10:1/1:2 ratio, when using the declared values for free sugars (true) and the predicted values (predicted). *p*-values refer to a *z*-test to compare proportions, *p* > 5% means that the difference between the proportions is not statistically significant.

The percentage of products meeting the carbohydrates ratio aggregating all categories ranged from 67% in UK to 9.8% in Malaysia. We should note that Chile, France, Germany, and the UK all have very small sample sizes which may not be representative of all products in the country.

In addition, we enumerated the products in each country with at least one of the following positioning claims given by Mintel GNPD: “High/Added Fibre,” “Wholegrain,” “Low/No/Reduced Carb,” “Low/No/Reduced Glycemic,” “Low/Reduced Sugar,” “No Added Sugar,” “Diabetic.” These represented Australia (31%), Brazil (19%), Chile (20%), France (15%), Mexico (12%), Nigeria (11%), Philippines (9%), Singapore (20%), South Africa (16%), Thailand (15%), UK (15%), of the products. Moreover, Australia had also the highest “Wholegrain” claims from all countries, with around 10% of products having the claim.

Table 2 illustrates the accuracy of the predictions, by comparing the predicted values with the declared ones; we then applied the predictive model to the full dataset, including the products where declared values for free sugars were not available. The results are shown in Table 3, for the same list of selected countries.

**Table 3:** Carbohydrate quality for selected countries across categories, using the full dataset, calculated after merging declared and predicted values.

Market	*n*	Carbohydrates (g/100 g)	Fibers (g/100 g)	Free sugars (g/100 g)	% Products meeting 10:1	% Products meeting 1:2	% Products meeting 10:1|1:2 ratio (declared + predicted)
Australia	3,695	44.7 ± 22.4	5.5 ± 4.9	11.7 ± 12.2	47.8	49.2	37.2
Brazil	3,557	48.3 ± 24.8	4.0 ± 4.5	13.7 ± 15.2	32	37.9	26.1
Chile	836	51.4 ± 22.5	5.8 ± 5.5	13.1 ± 13.5	42.6	49.2	36.6
France	13,421	52.3 ± 20.4	4.3 ± 3.6	16.2 ± 13.6	28.9	34.1	19.3
Germany	10,533	49.4 ± 20.6	5.4 ± 4.4	13.4 ± 13.6	44.6	47.1	33.7
India	5,863	55.9 ± 24.7	5.0 ± 5.5	13.4 ± 14.6	27.3	38	22.8
Malaysia	2,557	54.0 ± 27.1	4.0 ± 4.3	18.4 ± 15.2	22.6	21.1	13.7
Mexico	12,424	50.1 ± 26.8	3.7 ± 4.8	16.3 ± 15.2	24.3	26.9	17.3
Nigeria	2,742	59.6 ± 21.2	3.3 ± 3.7	20.9 ± 14.9	14.8	17	9.5
Philippines	3,091	53.9 ± 27.1	3.1 ± 4.0	20.5 ± 15.7	15.5	14	8.5
Singapore	2,850	52.0 ± 26.9	4.1 ± 5.4	18.3 ± 16.0	24.6	24.1	15.9
South Africa	1,588	55.5 ± 21.5	5.0 ± 5.4	18.1 ± 14.9	30.8	32.7	23.6
Thailand	5,456	46.2 ± 29.6	3.5 ± 5.2	14.1 ± 14.8	21.1	24.9	14.2
UK	17,589	50.6 ± 19.9	4.3 ± 4.2	16.7 ± 14.8	30	36.8	22.4
USA	33,809	52.5 ± 24.0	4.5 ± 4.7	16.6 ± 15.0	29.6	33.2	20.6

The countries are the same as in Table 2, *n* is the number of products for which the information was sufficient to apply the algorithm.

A table of accuracy results for all countries is provided in the Supplementary Table 3.

### Evolution of quality of carbohydrates over time

**Figure 4:**
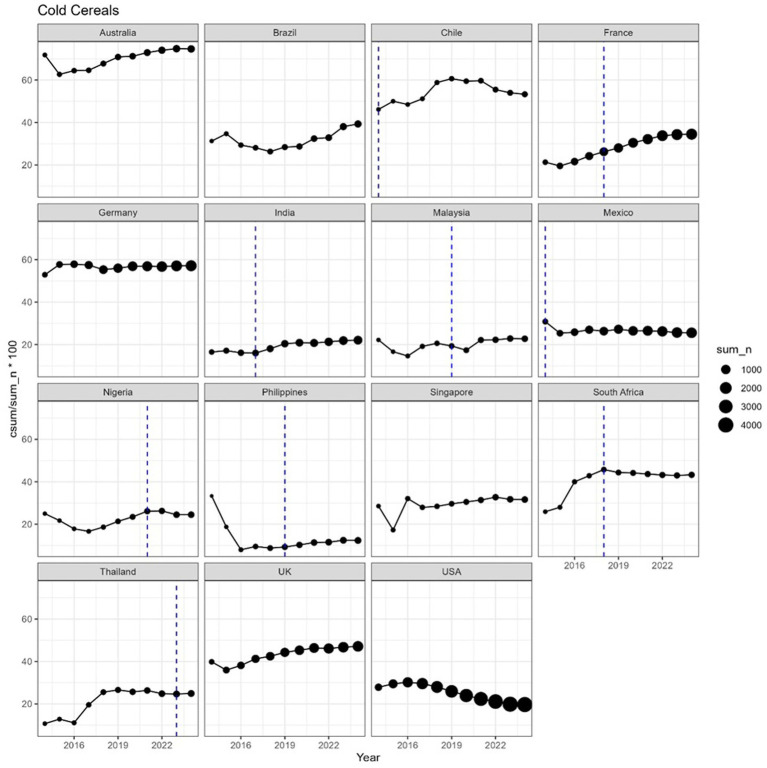
On the *y*-axis, the incremental proportion of products meeting the 10:1|1:2 ratio, for selected countries, during the period 2014–2024. Incremental means that, at year Y, the value in the graph is the % calculated cumulating all years up to Y. Vertical lines represent the year of introduction of taxes on sugar-sweetened beverages. The size of each dot is proportional to the number of products. Cold cereals are defined as any cereal (e.g., corn flakes, shredded wheat, toasted oat cereal) that is usually consumed dry or with dairy/non-dairy milk.

**Figure 5:**
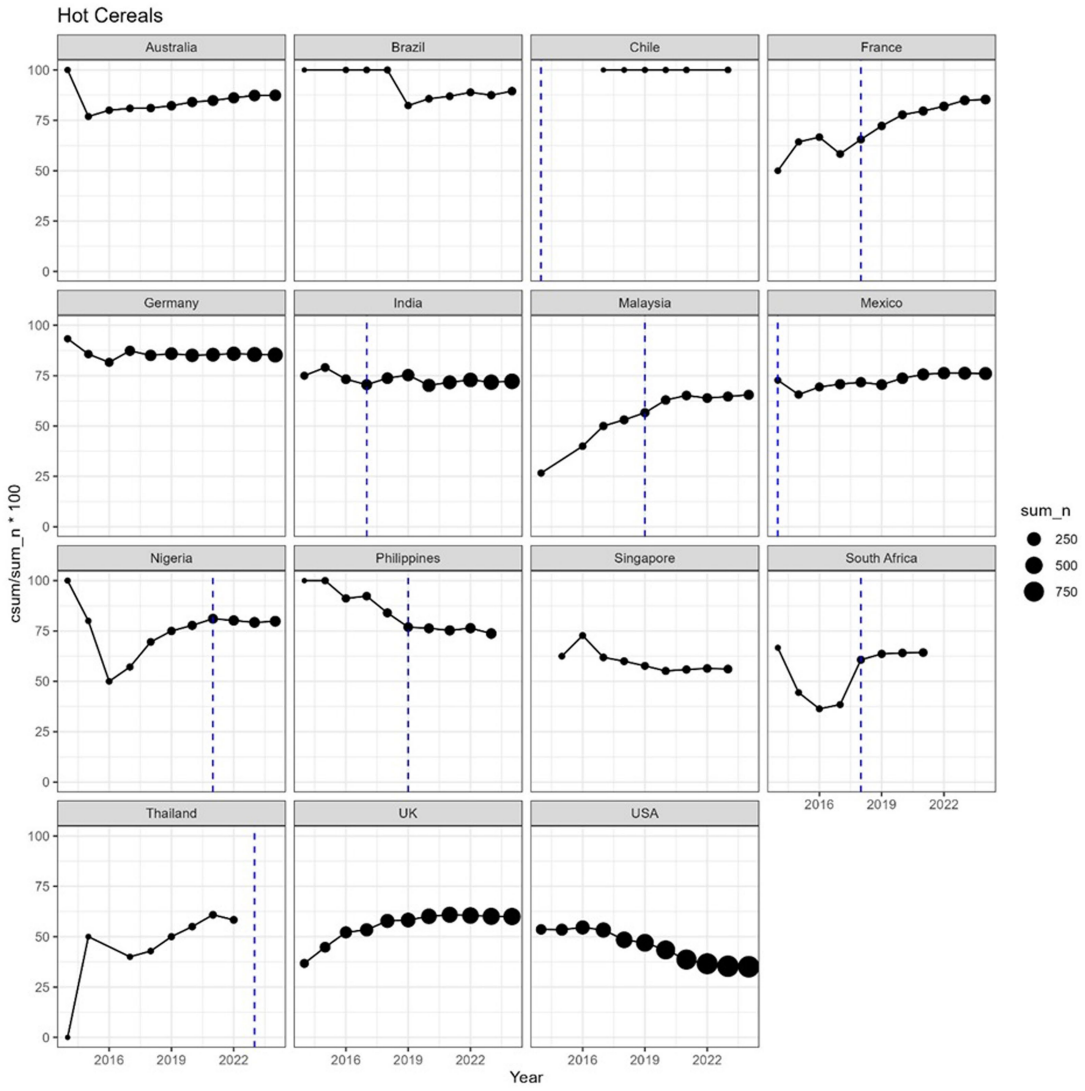
On the *y*-axis, the incremental proportion of products, meeting the 10:1|1:2 ratio, for selected countries, during the period 2014–2024. Incremental means that, at year Y, the value in the graph is the % calculated cumulating all years up to Y. Vertical lines represent the year of introduction of taxes on sugar-sweetened beverages. The size of each dot is proportional to the number of products. Hot cereals are defined as cereal that must be cooked (on the stovetop or in the microwave oven) before eating, including oatmeal, instant oatmeal, hot wheat, and other grain products.

The proportion of products meeting the 10:1|1:2 did not follow a consistent pattern in most countries, across all categories (see Figures 4, 5 for hot and cold cereals).

In general, we did not observe any significant change of the time trends corresponding to the introduction of a sugar tax (indicated as a vertical line in the figures). The number of products available varied widely between countries, so that a trend, or absence thereof, can be observed with higher confidence in countries where the number of products was consistently high in the period 2014–2023, for example France, Germany, Mexico or USA. For cereal products, a decreasing trend was observed in the USA starting from 2016–2017. In some cases, e.g., hot cereals in Thailand, the number of products was low (<10 in 2014–2018) and more data would be needed to make a reliable estimation of the carbohydrate quality in the food supply.

## Discussion

In this study, we developed and tested a method to predict the content of free sugars in packaged foods and applied it to monitor the adherence to a measure of carbohydrate quality globally, based on WHO recommendations. Carbohydrate quality was characterized by a ratio of total carbohydrates, dietary fibers and/or free sugars, that had previously been positively associated with better nutritional quality in Australia, US and Southeast Asia (28). Our approach to the prediction of free sugars consists of a combination of predictions from multiple machine learning models, using the Mintel Global New Product Database 2014–2024, based on information declared on pack, namely the list of ingredients and the declared values of energy, total fats, saturated fatty acids, carbohydrates, dietary fibers, total sugars, protein, and sodium.

Similar to previously published studies, our approach is based on machine learning models to predict the content of added/free sugars based on information found on product packaging (25, 26) that utilized. However, compared to these different methods, our novel approach has several key differentiating points: firstly, we conducted a thorough curation process to identify and categorize ingredient names that indicate the presence of free sugars as well as natural sugars from dairy, fruits, and vegetables. We utilized regular expressions to search for these specific ingredient names, allowing us to accurately identify and differentiate between different types of ingredients. Secondly, instead of relying on a single machine learning model, we employed a combination of predictions from multiple models. This approach enhances the flexibility and extensibility of our method. Our approach was based on state-of-the art machine learning techniques and can be re-run on each updated version of the database, as new or re-formulated products are launched. For the choice of the model, multiple algorithms were tested, and we found that, in general, tree-based models were the most effective; in particular kNN (“k-nearest neighbors”), used by Davies et al. (25), performed significantly worse than the models we selected.

In order to test the practical relevance of this method, we applied it to packaged food products of 77 countries where free sugars were not declared and estimated the carbohydrates quality of these products, reflected by the 10:1|1:2 carbohydrate ratios in. Results revealed that considerable variation in carbohydrate quality was observed between the different food categories, and within each category. In the US, the highest carbohydrate quality was observed for hot cereals, fruit snacks, cold cereals, and plant-based drinks. This is in line with results from Sievenpiper (5), although the percentages we found differ somewhat from theirs. On the other hand, the analysis in Liu et al. (15) was based on the USDA Food and Nutrient Database for Dietary Studies (FNDDS), which is a generic food composition table for dietary studies. FNDDS does include estimates for added sugars [through the Food Patterns Equivalent Database (20)] but does not include packaged food products in general; USDA makes available a large database of packaged products but information on added sugars is currently very limited. Overall, the accuracy of the predictions ranged from values close to the US (e.g., for Australia and UK), to errors more than three times bigger (for Thailand). For some countries, the estimate of the model accuracy may not be entirely reliable at this stage, given the small number of products and the under-representation of some categories. However, as the database grows over time, the model can be refined and re-run with minimal effort, given that the data pre-processing steps were automated. We observed marked differences between product categories: indeed, beverages had the lowest adherence to the 10:1|1:2 compared to solid foods, probably due to their lower fiber content (29, 30). Interestingly, only the plant-based beverages showed a relatively high adherence to the ratio, compared to other beverages. This could be explained by both a higher fiber content, as well as lower added sugar level. Indeed, as most of these beverages may be consumed as dairy substitutes, the target levels of added sugars for this category were set to ideally (31) match the level of naturally occurring sugar in milk (around 5 g/100 g), with a target for “best of class” products of 2.65 g/100 g for children. Our estimate was of 2 g/100 g of free sugars on average, consistent with this general guidance. Accordingly, in Drewnowski et al. (31), it was estimated that 73.8% of the plant-based beverages in the USDA branded products database had less than 2.65 g of added sugars per 100 g.

When comparing the carbohydrates quality between different countries using our algorithm, we observed a high variability. For example in Australia around 40% of products met the 10:1|1:2 criteria across all categories (*N* = 10,659), while only around 15% of the products qualified in Brazil, Nigeria and the Philippines. In fact, Australia was found to have the largest proportion of products that met the 10:1|1:2 criteria, extending previous observations (17, 28), where Australia was compared to several South-East Asian countries. This observation is supported by the fact that, in the Mintel database, Australia has the highest proportion of products with claims on whole grains, and on other characteristics related to carbohydrate quality, such as “low glycemic” or “high fiber.”

Thanks to our methodology, we could confidently estimate the adherence to carbohydrate quality at a global scale, including countries with limited data. This is the first study, to our knowledge, to assess systematically the carbohydrate quality of packaged foods at such a scale. One notable exception was Russia, where we were not able to apply our algorithm because only energy, fat and protein tend to be declared (32). The algorithm needs presence of 8 nutrients (energy, fat, saturated fat, carbohydrates, sugars, fibers, protein, sodium) to successfully predict carbohydrate quality, which is not mandatory in every country’s nutrient labelling regulations.

Our analysis showed a general decrease of carbohydrate quality, at least for cereals, since 2016. However, from 1999 to 2016, a significant decrease in percentage of energy intake from low-quality carbohydrate was observed among adults (33). Additionally, in the United States, a decrease in added sugars intake was observed among younger adults (19–50 years) from 2001 to 2018 (33), possibly related to public health policies targeting sugar-sweetened beverages; product reformulations and new product launches to reduce the added sugars content in sweetened beverages may also have contributed. We are not aware of a similar analysis of intake data for the period after 2016, it would be interesting to see whether the carbohydrate quality of new product launches aligns with intake trends.

Our approach based on machine learning models may be used in other applications than enabling the calculation of carbohydrate quality. Previously, the authors have developed automated methods to estimate free sugars in food composition tables (24). Combining databases of non-branded and branded foods will provide a comprehensive assessment of the carbohydrate quality in the food supply at a global scale, filling a major gap in public health research. Furthermore, these models are applicable more generally to any food or nutrient metric that requires free sugars as an input such as nutrient profiling models. For instance, the development of nutrient profiling models for application in low- and middle-income countries’ such as the Nutrient Rich Food Index, a nutrient density metric featuring added sugars (34), is limited by food composition data availability and could benefit from the prediction of our machine learning models. As a further example, a recent publication (35) highlighted that the current version of the Nutri-Score relies on a component for total sugars, as it is the only available information on the back-of-pack. However, from a public health perspective, added sugars or free sugars are more relevant for health outcomes than total sugars (36). The introduction of free sugars in the Nutri-Score algorithm may allow for a subtler discrimination between products. The present work evaluated the carbohydrates quality of packaged food products using the carbohydrates ratio. Recently, alternative metrics for carbohydrate quality have been proposed, building on the existing ones. For example, Drewnowski et al. (8) introduced a new carbohydrate food scoring system, which supplements the fiber and free sugar components of previous metrics with additional dietary components of public health concern (e.g., sodium, potassium, and whole grains) as identified by the Dietary Guidelines for Americans. As these more recent metric also include free sugars as component, our work may be applied to automate the calculation of such an extend score as well.

Among the strengths of this study, we would like to emphasize the fully automated approach, including a thorough data curation with a list of regular expressions that was manually curated and may be enriched whenever necessary. The method is fully automated and can be easily updated as new products are added to the database. As an example, while we chose to use Mintel’s GNPD for our study based on its geographical reach, our method may also be used to predict free sugars content in other databased such as the USDA’s branded products database. The predicted values for free sugars were used to predict the ratio of carbohydrate to free sugar, thus enabling us to estimate carbohydrate quality of the products. Since our definition of carbohydrate quality is based on a threshold for this ratio, predictions with a high error for free sugars did not penalize the results for carbohydrate quality; in fact, our method demonstrated great accuracy in predicting carbohydrate quality in multiple categories and countries.

The algorithm could be implemented in a digital tool (e.g., a smartphone app), reading the ingredient list and the nutrient information directly from a picture of a product. In the absence of explicit information available on the food labels, the algorithm may also be used by industries or health organizations to monitor the carbohydrate quality in the manufactured food supply, its evolution over time and the effectiveness of regulations or of front-of-pack labelling interventions.

Among the limitations, we acknowledge the assumption that free sugars equals added sugars in the selected product categories and the fact that the definition of what exactly counts as added sugars might vary slightly between countries. In addition, rules for rounding and tolerances in food labels tend to be country-specific, but our model at this point did not account for this. Additionally, in countries where the declaration of added or free sugars is not mandatory, the products declaring them might be a biased sample; we observed a difference in the carbohydrate and fiber content in products sold in Australia, where products not declaring added sugars had a higher content of carbohydrates and lower content of fibers.

This is the first study, to our knowledge, predicting added and free sugars and carbohydrate quality at a large scale. Carbohydrate quality was shown to vary across countries and within packaged food categories. Although we focused on 15 countries for simplicity of presentation, we applied our algorithm to 77 countries on a database spanning almost 10 years, thus creating a database that can serve as a basis for further analyses.

## Data Availability

The data analyzed in this study is subject to the following licenses/restrictions: GNPD is a commercial dataset produced and distributed by Mintel. Requests to access these datasets should be directed to https://mintel.com. Interested parties are welcome to contact Eric Scuccimarra for permission to access the computer codes used in this study for use in collaborative projects.
