# Supplementary material for: Predicting carbohydrate quality in a global database of packaged foods
Source: Front Nutr. 2025 Mar 12;12:1530846. doi: 10.3389/fnut.2025.1530846 (PMC11936779; doi:10.3389/fnut.2025.1530846)
Supplement: Supplementary file 1 [file Data_Sheet_1.docx]

Supplementary Material

# Prediction methodology

## Data cleaning

Globally, 2,412,463 non-duplicate products were provided by Mintel, launched from January 2014 to February 2024. 152,333 (6.3%) contain information for “added sugar”, of which 151,684 had a retrievable value (99.6% of products declaring added sugars). If sodium was missing, salt was translated into sodium (sodium = 1000*salt/2.5). We excluded 379,689 products missing information for serving size or where the serving measure was not declared in “g”, “ml”, “fl. oz (US)”, or “oz”. In addition, 1,087,245 products were dropped as at least one of the nutrients used as predictors in the machine learning model - total carbohydrates, total sugars, protein, total fats, sodium, dietary fibers – was missing and the value using could not be imputed via the back of pack or with simple rules (e.g. if total fat is 0, total saturated fat is also 0). Finally, 57,954 products were removed as they contained implausible nutrient values (e.g. the sum of all nutrient values is greater than 100g per 100g). The final dataset contained a total of 887,575 products (36.8% of raw dataset) with 123,035 products declaring added sugars (69,644 of which were from USA, consisting of 56.6% of the final set products declaring added sugars).

## Ingredient tagging

Food labels are generally required to list ingredients in descending order by quantity, and Mintel GNPD includes this information. On-pack ingredients can be complex to parse, with some ingredients containing their own list of sub-ingredients, some of which may, in turn, contain their own sub-ingredients. We split the list of ingredients into individual ingredients, ensuring that sub-ingredients are properly included, and then examined the list of ingredients to determine whether each was or contained added sugars, as well as dairy or fruits and vegetables, which were considered to be the major sources of natural sugars.

Due to the complexity of ingredients, simple string matching would incorrectly identify many ingredients. For example, “sugar” should be considered added sugar ingredient, but “sugar-free”, “sugar-peas”, and “may contain sugar” should not. To address this, we compared each ingredient to a set of regular expressions. The regular expressions for added sugars were based on Mintel’s list of added sugar ingredients and were further refined by applying them to the data and reviewing the results to identify products which contained added sugar but had no identified added sugar ingredients, or vice versa. The complete list of regular expressions for added sugars is provided in Supplementary table 1. Similar expressions were used to identify the dairy and fruit/vegetable ingredients, which are likely to contain natural sugars.

This method was applied, for each of the three tags, to the first 15 ingredients with binary columns being added to the data. The first 6 ingredient tags were used as features for the models, as well as an additional binary column indicating if any of the 15 ingredients were considered added sugar ingredients.

## Labeling tolerance

In the US, nutrition data is reported per serving and added sugars can be rounded to the nearest integer or rounded down to 0 if less than 1 gram per serving. This results in a potential difference between actual and declared added sugars of up to 0.5g/serving or 1g/serving for products which declare 0 grams. As we consider all nutrition per 100 grams, this difference can become significant for products with small serving sizes. To address this issue, we have established a tolerance range for each product based on its serving size. The tolerance range is used to adjust the error, as described below.

We found that many products included added sugar ingredients but declared 0 grams of added sugars. Since we are unsure whether this is due to labelling errors or rounding down, we removed these products from the dataset to avoid training on potentially erroneous data. Since products declaring 0 total sugars cannot contain any added sugars, any such products were removed by the training data as this case can be addressed by a simple rule.

## Algorithm development

We developed a multi-step prediction model, as outlined in Figure 2 in the main text: first multiple tree-based regression models were fit to predict the amounts of added sugars per 100g. Those regression models were then combined, using a no-intercept positive coefficients only linear model, resulting in a weighted average of the models. The regression models used are random forest, extra trees, and Ada boost with extra trees as estimators.

Each regression model is fit twice, once to predict the actual added sugar, and a second time to predict the ratio of added sugar to total sugar, which is then multiplied by total sugar to yield predicted added sugar. Models which directly predict the added sugars at times predict greater added sugars than total sugar, to address this all predictions are capped at the amount of total sugars.

We found that the regression models were largely unable to identify which products contained no added sugars, rarely predicting values of 0. To address this we added a separate binary classifier to predict whether a food did or did not contain added sugars. The classifier consisted of three tree-based classifiers, with the final prediction determined by majority vote. Products which were predicted to contain no added sugars had the predictions of the regression models overridden to 0. The classification models used are random forest, gradient boosting machine, Ada boost with extra trees as the estimators.

As predictors for both the regression and classification models, we used binary variables indicating whether each of the top six ingredients was tagged as containing added sugars, dairy, or fruit, and the amounts of the following nutrients: energy (kcal/100g), total fats (g/100g), SFA (g/100g), total carbohydrates (g/100g), dietary fibers (g/100g), total sugars (g/100g), protein (g/100g), sodium (mg/100g). The choice of these nutrients was suggested by the fact that their declaration on pack is mandatory in most countries and therefore their values are usually available, independently of the category and the market. The decision to use the top six ingredients was made as that provided the best performance on the validation set.

A separate prediction model was fit for each Mintel GNPD category. For each category, the products in that category were randomly split into 75% of samples to be used as a training set, with the remaining samples used as a validation set to test the accuracy of the predictions. The split was stratified to ensure that the proportion of products that did and did not contain added sugars was the same in the training and test sets.

When fitting the regression models, the training dataset was again split, with 70% used to fit the regression models, with the remaining 30% used to fit the stacking linear model with the output of the regressors as predictors. The full training set was used to fit the voting classifier.

## Measure of accuracy and validity

To assess the accuracy of the regression models we employed a modified version of Mean Absolute Error (MAE) which accounted for the labeling tolerance as described above. For each product, if the predicted value of added sugars fell within the defined tolerance range, the MAE was set to 0, indicating a correct prediction of added sugars. However, if the predicted value was outside the tolerance range, the MAE was calculated as the absolute difference between the declared added sugars value and the predicted value. This approach allowed us to evaluate the accuracy of the regression models while considering the defined tolerance range for added sugars. Accuracy and F1 score were used to measure the performance of the classifiers.

Finally, we tested the predictions on the products from all countries, under the implicit assumption that the predictability of added sugars from the information on label should not be specific to the US data.

## Results

When applying models trained on US data to the 81 other countries for which there was sufficient data, the result is an overall MAE of 2.05 and 58% of predictions within the US tolerance range. The models performed best on Ghana (MAE = 0.6) and Panama (MAE = 0.82) and worst on Vietnam (MAE = 3.48) and Thailand (MAE = 3.86).

Applying Davies’ method to our dataset, using the same nutrients used by our models as starch, which is used by Davies, is typically not declared on labels, resulted in an MAE of 2.51. If we apply the same kNN method including the binary columns for ingredient tagging the MAE is 2.45. Applying the nutrient-only kNN method to the other countries in our dataset results in an MAE of 4.53.

# Supplementary Figures and Tables

|  | **Ingredient** | **Total** |
| --- | --- | --- |
| (?<!salsa.*)(?!cured with.*)(?<!may contain.*)(?<!bacon \(.*)(?!.*seasoning.*)(?<!sriracha sauce.*)(?<!cottage cheese.*)sugar(?!.* free\|-free\| peas\| snap peas) | sugar | 97408 |
| (agave\|maple\|cane\|rice\|corn\|date\|tapioca\|maltitol\|coconut\|coconut palm) syrup | syrup | 19480 |
| (?<!may contain.*)(?<!bacon \(.*)(?<!^malt .*)(?<!cultured\W)(?<!cottage cheese.*)dextrose | dextrose | 10194 |
| (?<!may contain.*)glucose | glucose | 8597 |
| (?<!may contain.*)fructose | fructose | 7854 |
| maltodextrin$\|^maltodextrin | maltodextrin | 6033 |
| (?<!bacon \(.*)honey | honey | 5026 |
| (?<!juice \(.*)(cherry\|pear\|apple\|grapefruit\|grape\|acerola\|apricot\|aronia\|bilberry\|blackberry\|blackcurrant\|blood orange\|berry\|kiwi\|guava\|jackfruit\|lychee\|mango\|orange\|papaya\|passion fruit\|peach\|pear\|plum\|pomegranate\|pomelo\|prune\|raisin\|soursop\|tangerine\|must) juice concentrate | juice concentrate | 4217 |
| (?<!may contain.*)lactose(?!.* free\|-free) | lactose | 3578 |
| molasses | molasses | 3257 |
| caramel\s(?!.*color) | caramel | 2642 |
| (?<!may contain.*)sucrose(?!.*esters) | sucrose | 1349 |
| agave(?!.*inulin) | agave | 610 |
| (?<!juice \(.*)(cherry\|pear\|apple\|grapefruit\|grape\|acerola\|apricot\|aronia\|bilberry\|blackberry\|blackcurrant\|blood orange\|berry\|kiwi\|guava\|jackfruit\|lychee\|mango\|orange\|papaya\|passion fruit\|peach\|pear\|plum\|pomegranate\|pomelo\|prune\|raisin\|soursop\|tangerine\|must) concentrate | fruit concentrate | 571 |
| (?<!100% .*)(?<!fruit juice \()concentrated (cherry\|pear \|apple\|grapefruit\|grape\|acerola\|apricot\|aronia\|bilberry\|blackberry\|blackcurrant\|blood orange\|berry\|kiwi\|guava\|jackfruit\|lychee\|mango\|orange\|papaya\|passion fruit\|peach\|pear\|plum\|pomegranate\|pomelo\|prune\|raisin \|soursop\|tangerine\|must) juice | concentrated juice | 483 |
| jaggery | jaggery | 228 |
| (concentrated) grape must | concentrated grape must | 188 |
| coconut \w+ nectar\|coconut nectar | coconut nectar | 153 |
| cane juice | cane juice | 139 |
| balsamic vinegar of modena | balsamic vinegar of modena | 68 |
| piloncillo | piloncillo | 37 |
| concentrate \w+ juice | concentrate juice | 12 |

*Supplementary table 1. List of regular expressions used to identify automatically ingredients susceptible to being considered as source of added sugars*

|  | **n** | **True** | **Pred** | **R2** | **MAE** |
| --- | --- | --- | --- | --- | --- |
| **Baby Food** | 200 | 4.3 ± 9.5 | 4.1 ± 8.6 | 0.89 | 0.8 |
| **Bakery** | 2249 | 24.2 ± 17.2 | 24.1 ± 16.7 | 0.96 | 1.31 |
| **Breakfast Cereals** | 574 | 19.6 ± 11.3 | 19.3 ± 10.9 | 0.97 | 0.88 |
| **Chocolate Confectionery** | 873 | 41.6 ± 12.0 | 41.4 ± 11.3 | 0.88 | 1.71 |
| **Dairy** | 817 | 5.2 ± 9.9 | 4.5 ± 8.2 | 0.97 | 0.4 |
| **Desserts & Ice Cream** | 606 | 20.9 ± 14.6 | 21.5 ± 15.3 | 0.97 | 1.36 |
| **Fruit & Vegetables** | 690 | 0.9 ± 3.5 | 1.1 ± 4.0 | 0.96 | 0.17 |
| **Hot Beverages** | 86 | 47.8 ± 19.3 | 48.9 ± 18.9 | 0.91 | 2.18 |
| **Juice Drinks** | 169 | 2.4 ± 4.1 | 2.3 ± 4.0 | 0.96 | 0.29 |
| **Meals & Meal Centers** | 986 | 1.8 ± 2.7 | 2.0 ± 2.6 | 0.88 | 0.44 |
| **Nutritional Drinks & Other Beverages** | 158 | 13.3 ± 22.7 | 12.2 ± 23.1 | 0.98 | 0.87 |
| **Processed Fish, Meat & Egg Products** | 416 | 2.6 ± 3.3 | 2.3 ± 2.8 | 0.94 | 0.28 |
| **RTDs** | 74 | 5.1 ± 2.9 | 4.9 ± 2.7 | 0.94 | 0.3 |
| **Sauces & Seasonings** | 929 | 9.8 ± 13.7 | 9.7 ± 13.3 | 0.96 | 0.89 |
| **Savoury Spreads** | 157 | 1.8 ± 4.9 | 1.7 ± 5.1 | 0.95 | 0.26 |
| **Side Dishes** | 575 | 0.5 ± 1.7 | 0.6 ± 1.6 | 0.91 | 0.12 |
| **Snacks** | 2146 | 10.7 ± 13.9 | 10.4 ± 13.2 | 0.94 | 1.2 |
| **Soup** | 227 | 1.1 ± 3.5 | 1.3 ± 4.0 | 0.83 | 0.47 |
| **Sugar & Gum Confectionery** | 350 | 56.8 ± 16.7 | 57.0 ± 16.2 | 0.92 | 2.18 |
| **Sweet Spreads** | 280 | 20.5 ± 22.5 | 20.3 ± 21.5 | 0.97 | 1.33 |

*Supplementary table 2. Accuracy of the predictions for added sugars in the US test set, using the original Mintel categories.*

|  | **n** | **True** | **Pred** | **R2** | **MAE** |
| --- | --- | --- | --- | --- | --- |
| **Argentina** | 1156 | 20.6 ± 19.9 | 20.1 ± 19.6 | 0.93 | 1.91 |
| **Australia** | 335 | 8.5 ± 14.9 | 8.7 ± 15.0 | 0.98 | 0.84 |
| **Bangladesh** | 323 | 16.4 ± 20.0 | 16.5 ± 19.6 | 0.95 | 2.01 |
| **Belgium** | 54 | 10.9 ± 14.6 | 11.1 ± 15.2 | 0.93 | 1.37 |
| **Brazil** | 2498 | 16.2 ± 19.0 | 16.7 ± 19.1 | 0.94 | 1.71 |
| **Cambodia** | 333 | 17.5 ± 17.4 | 18.6 ± 17.8 | 0.92 | 2.47 |
| **Cameroon** | 111 | 16.0 ± 20.2 | 16.3 ± 19.4 | 0.93 | 2 |
| **Canada** | 185 | 17.0 ± 22.3 | 17.2 ± 21.9 | 0.97 | 1.86 |
| **Colombia** | 2529 | 17.7 ± 20.9 | 17.7 ± 20.6 | 0.94 | 1.86 |
| **Costa Rica** | 528 | 16.0 ± 18.7 | 16.1 ± 18.6 | 0.94 | 1.34 |
| **Egypt** | 512 | 23.8 ± 18.5 | 23.4 ± 18.6 | 0.85 | 2.4 |
| **Ethiopia** | 213 | 15.6 ± 19.7 | 16.1 ± 18.6 | 0.9 | 2.48 |
| **Ghana** | 138 | 13.0 ± 17.3 | 13.2 ± 17.3 | 0.99 | 0.6 |
| **Guatemala** | 315 | 16.6 ± 20.4 | 16.7 ± 20.6 | 0.97 | 1.25 |
| **Hong Kong, China** | 138 | 12.3 ± 17.0 | 12.8 ± 17.4 | 0.95 | 1.38 |
| **India** | 3025 | 10.6 ± 16.1 | 11.3 ± 16.6 | 0.88 | 2.12 |
| **Indonesia** | 227 | 15.8 ± 16.8 | 16.1 ± 16.9 | 0.93 | 1.94 |
| **Jordan** | 552 | 15.9 ± 20.1 | 15.8 ± 19.9 | 0.96 | 1.37 |
| **Kenya** | 146 | 23.2 ± 22.3 | 22.9 ± 22.1 | 0.97 | 1.83 |
| **Kuwait** | 627 | 16.4 ± 19.5 | 17.0 ± 19.7 | 0.93 | 2.05 |
| **Lebanon** | 227 | 14.4 ± 19.6 | 14.8 ± 19.5 | 0.9 | 2.06 |
| **Malaysia** | 579 | 13.5 ± 16.1 | 15.5 ± 17.5 | 0.87 | 3 |
| **Mexico** | 11067 | 14.2 ± 19.1 | 14.3 ± 18.6 | 0.94 | 1.73 |
| **Morocco** | 155 | 22.5 ± 20.1 | 22.9 ± 20.2 | 0.85 | 2.46 |
| **Myanmar** | 208 | 13.3 ± 14.9 | 14.8 ± 16.1 | 0.88 | 2.61 |
| **Netherlands** | 620 | 7.5 ± 14.1 | 7.7 ± 13.5 | 0.98 | 0.87 |
| **Nigeria** | 536 | 15.9 ± 17.7 | 17.1 ± 18.0 | 0.82 | 2.59 |
| **Oman** | 541 | 18.9 ± 20.4 | 19.3 ± 20.6 | 0.9 | 2.32 |
| **Pakistan** | 237 | 17.2 ± 18.4 | 17.7 ± 18.1 | 0.93 | 1.89 |
| **Panama** | 656 | 14.8 ± 18.8 | 14.8 ± 18.6 | 0.98 | 0.82 |
| **Peru** | 410 | 13.6 ± 17.3 | 13.9 ± 17.2 | 0.93 | 1.91 |
| **Philippines** | 746 | 19.7 ± 20.1 | 20.7 ± 20.2 | 0.93 | 2.21 |
| **Puerto Rico** | 2692 | 15.9 ± 18.1 | 15.9 ± 18.0 | 0.98 | 0.95 |
| **Qatar** | 541 | 19.9 ± 20.0 | 21.0 ± 20.5 | 0.87 | 2.63 |
| **Saudi Arabia** | 986 | 17.1 ± 21.1 | 18.1 ± 21.2 | 0.92 | 2.34 |
| **Singapore** | 941 | 17.7 ± 18.7 | 18.6 ± 18.9 | 0.94 | 2.03 |
| **South Africa** | 195 | 22.9 ± 19.6 | 23.8 ± 19.9 | 0.92 | 1.92 |
| **South Korea** | 80 | 13.5 ± 21.2 | 13.4 ± 20.3 | 0.96 | 1.37 |
| **Sri Lanka** | 227 | 14.0 ± 16.8 | 14.2 ± 16.7 | 0.87 | 2.32 |
| **Tanzania** | 206 | 14.6 ± 18.0 | 15.3 ± 18.9 | 0.82 | 2.22 |
| **Thailand** | 156 | 11.2 ± 16.1 | 13.4 ± 18.0 | 0.74 | 3.86 |
| **Tunisia** | 208 | 23.5 ± 18.3 | 24.3 ± 18.3 | 0.89 | 2.87 |
| **UAE** | 1054 | 17.4 ± 19.9 | 17.8 ± 20.1 | 0.92 | 2.07 |
| **UK** | 91 | 17.5 ± 18.5 | 18.0 ± 19.0 | 0.98 | 1.2 |
| **Venezuela** | 256 | 12.9 ± 18.9 | 12.7 ± 18.5 | 0.98 | 1.01 |
| **Vietnam** | 549 | 15.8 ± 16.9 | 18.0 ± 18.1 | 0.77 | 3.48 |

*Supplementary table 3 Accuracy of added sugar predictions on countries outside US, non-category specific – excluding countries with n < 50*

|  | **n** | **kNN R2** | **kNN MAE** | **Our R2** | **Our MAE** | |
| --- | --- | --- | --- | --- | --- | --- |
| **Baby Food** | 200 | 0.74 | 1.39 | 0.89 | 0.8 |  |
| **Bakery** | 2249 | 0.89 | 3.27 | 0.96 | 1.31 |  |
| **Breakfast Cereals** | 574 | 0.85 | 2.47 | 0.97 | 0.88 |  |
| **Chocolate Confectionery** | 873 | 0.78 | 3.24 | 0.88 | 1.71 |  |
| **Dairy** | 817 | 0.79 | 0.99 | 0.97 | 0.4 |  |
| **Desserts & Ice Cream** | 606 | 0.88 | 2.85 | 0.97 | 1.36 |  |
| **Fruit & Vegetables** | 690 | 0.7 | 0.56 | 0.96 | 0.17 |  |
| **Hot Beverages** | 86 | 0.76 | 4.86 | 0.91 | 2.18 |  |
| **Juice Drinks** | 169 | 0.6 | 1.46 | 0.96 | 0.29 |  |
| **Meals & Meal Centers** | 986 | 0.34 | 1.22 | 0.88 | 0.44 |  |
| **Nutritional Drinks & Other Beverages** | 158 | 0.87 | 3.22 | 0.98 | 0.87 |  |
| **Processed Fish, Meat & Egg Products** | 416 | 0.34 | 1.45 | 0.94 | 0.28 |  |
| **RTDs** | 74 | 0.79 | 0.82 | 0.94 | 0.3 |  |
| **Sauces & Seasonings** | 929 | 0.76 | 3.31 | 0.96 | 0.89 |  |
| **Savoury Spreads** | 157 | 0.28 | 1.7 | 0.95 | 0.26 |  |
| **Side Dishes** | 575 | 0.36 | 0.44 | 0.91 | 0.12 |  |
| **Snacks** | 2146 | 0.77 | 3.42 | 0.94 | 1.2 |  |
| **Soup** | 227 | 0.53 | 1.02 | 0.83 | 0.47 |  |
| **Sugar & Gum Confectionery** | 350 | 0.81 | 4.06 | 0.92 | 2.18 |  |
| **Sweet Spreads** | 280 | 0.88 | 3.88 | 0.97 | 1.33 |  |

*Supplementary table 4. Comparison of our method with Davies kNN method applied using the features available in Mintel, by Mintel category*

**
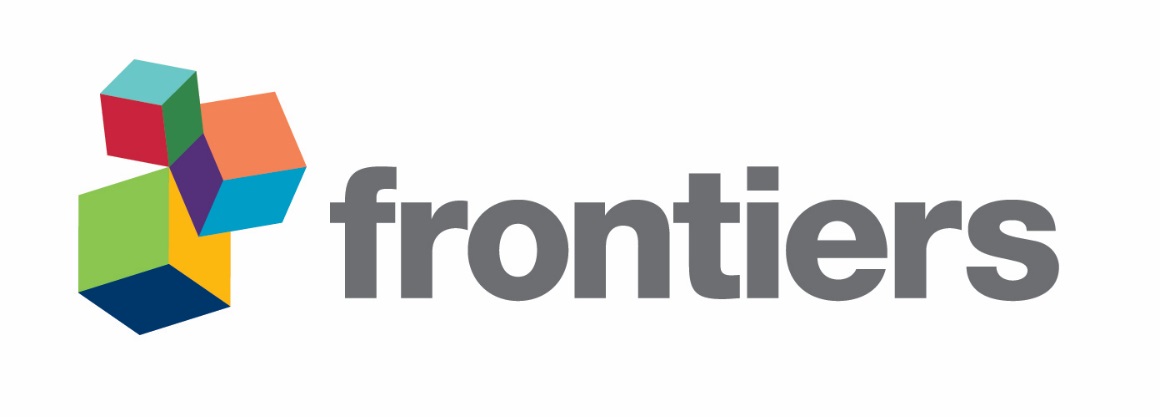
**
